# ORFeome-based identification of biomarkers for serodiagnosis of *Mycobacterium tuberculosis* latent infection

**DOI:** 10.1186/s12879-017-2910-y

**Published:** 2017-12-28

**Authors:** Fangbin Zhou, Xindong Xu, Sijia Wu, Xiaobing Cui, Weiqing Pan

**Affiliations:** 1 Institute for Infectious Diseases and Vaccine Development, Tongji University School of Medicine, Shanghai, People’s Republic of China; 2 Department of Oncology, The Second Clinical Medical College, Shenzhen People’s Hospital, Jinan University, Shenzhen, People’s Republic of China; 3 Department of Tropical Infectious Diseases, Second Military Medical University, Shanghai, People’s Republic of China

**Keywords:** *Mycobacterium tuberculosis*, Latent TB infection, Serodiagnosis, Recombinant GST-TB fusion proteins, Sensitivity and specificity

## Abstract

**Background:**

The challenges posed by *Mycobacterium tuberculosis* infection require the gradual removal of the pool of latent tuberculosis infection (LTBI). The current cell-immune-based diagnostic tests used to identify LTBI individuals have several irreversible drawbacks. In the present study, we attempted to identify novel diagnostic antigens for LTBI.

**Methods:**

A high-throughput glutathione S-transferase (GST)-fusion technology was used to express over 409 TB proteins and sera from LTBI and healthy individuals was used to interrogate these GST-TB fusion proteins.

**Results:**

Of 409 TB proteins, sixty-three reacted seropositive and defined the immuno-ORFeome of latent *M. tuberculosis.* Within the immuno-ORFeome, the rare targets were predominantly latency-associated proteins and secreted proteins, while the preferentially recognized antigens tended to be transmembrane proteins. Six of novel highly-reactive antigens had the potential to distinguish LTBI from active TB and healthy individuals. A multiple-antigen combination set was selected through analysis of various combinations. A panel of 94 archived serum samples was used to validate the diagnostic performance of the multiple-antigen combination set, which had sensitivity of 66.1% (95% CI 52.9, 77.4) and specificity of 87.5% (95% CI 70.1, 95.1).

**Conclusion:**

These results provide experimental evidence of the immunogenicity of novel TB proteins that are suitable for the development of serodiagnostic tools for LTBI.

**Electronic supplementary material:**

The online version of this article (10.1186/s12879-017-2910-y) contains supplementary material, which is available to authorized users.

## Background

Tuberculosis (TB) is the second leading cause of death from infectious disease worldwide, infecting approximately 10.4 million individuals and leading to 1.4 million deaths in 2015 [1]. However, until now, tuberculosis control has mainly concentrated on detection, treatment and management of active disease, which may not benefit the goal of global elimination. The challenges posed by *Mycobacterium tuberculosis* infection require a renewed focus on the gradual removal of the pool of latent tuberculosis infection (LTBI) present in some 2 billion people, an estimated 30% of the global population [2], which will be critical to future progress. LTBI is defined as a state of persistent immune reactivity to *M. tuberculosis* antigenic challenge in a healthy subject without evidence of clinically manifested active TB [3]. It is generally considered that 5% to 10% of infected individuals have no signs or symptoms of TB but are in danger of developing active TB during their lifetime. These groups include people living with HIV, recently infected persons, adult and child contacts of pulmonary TB cases, and tumor necrosis factor (TNF) and transplantation patients [4].

As the pathogen *M. tuberculosis* can only be isolated from humans with active disease, a direct measurement tool for LTBI is currently unavailable and the detection of LTBI is totally reliant on indirect measurements of immune response to stimulation by *M. tuberculosis* antigens. Of those, the in vivo, century old tuberculin skin test (TST) and the ex vivo, more recently available interferon-γ release assays (IGRAs) both only recognize an adaptive immune response to, but not necessarily a latent infection with, *M. tuberculosis* [3]. The former test is limited in use by its low specificity because of cross-reactivity with previous bacilli Calmette-Guérin (BCG) vaccination and other environmental mycobacteria. The IGRA, despite high specificity compared with TST, is expensive and needs particular expertise, which presents a barrier in resource-limited settings. Moreover, neither of these immune-based tests performs with optimal sensitivities in individuals who are immunocompromised or is able to discriminate active or past disease from latent infection, which is important in areas with a high burden of TB [4–6].

While it is well-recognized that T-cell-mediated immunity plays a central role in controlling the proliferation of *M. tuberculosis* and that IFN-γ is the predominant cytokine that indicates infection, other cytokines such as TNF-α, IP-10 and IL-2 have been investigated for their potential to enhance the diagnostic performance of IGRAs [7–9]. Moreover, although little is known about using B cells and humoral immunity for alternative diagnostic measurements for LTBI, mainly because *M. tuberculosis* is an intracellular bacterium, it appears that antibody-based B cells can encounter antigens in ectopic B cell aggregates associated with tuberculous granulomas [10], and several studies have shown dormancy-associated antigens do have the ability to discriminate latently infected individuals from healthy subjects or active tuberculosis patients [11–13]. In addition, several nutrient starvation or hypoxia models simulating latent *M. tuberculosis* infection revealed protein upregulation during adaptation to low-oxygen or nutrient deficit dormancy [14–16], providing evidence for a number of potential antigen targets as biomarkers of latent TB. Recently, a cocktail of DosR-regulon-encoded antigens (latency antigens) were evaluated for their immunogenicity; however, the value of this work was limited by the small number of antigens used [17].

The availability of *M. tuberculosis* genome sequence information along with corresponding proteomic datasets make possible the comprehensive, systematic and unbiased identification of novel antigenic proteins at the whole proteome scale [18]. Recently, we developed a glutathione S-transferase (GST) fusion protein assay at the ORFeome scale, which has been successfully applied to identify diagnostic markers for active tuberculosis and other pathogens [19, 20]. This technique integrates multiple advantages as follows: high throughput screening, faster and less labor-intensive, potential identification of the protein markers at low expression at nature condition and correct refolding of the GST fusion proteins. Although these novel diagnostic protein markers will provide support for the TB control program in achieving a reduction in the transmission of this disease, it is noteworthy that most of infected individuals have no symptoms of TB but are at high risk of developing active TB during their lifetime, which emphasizes the importance and urgency of the identification of diagnostic markers that can discriminate LTBI from healthy individuals. Thus, in the present study, as the extension of the previous study, we used this technology to generate over 400 latency-associated, transmembrane, secreted and region of difference (RD) proteins, which we interrogated using a large number of serum samples from LTBI individuals. We discovered several novel diagnostic antigens and used a panel of 94 archived serum samples from LTBI and healthy individuals to validate the diagnostic performance of a multiple-antigen combination set that provided a higher sensitivity and specificity, which may be beneficial for tracking LTBI.

## Methods

### Study population

This study was conducted with approval of the Internal Review Board, Tongji University School of Medicine, China. Written informed consent was obtained from the subjects. Ten LTBI patient serum samples for initial screening were collected from the staff from Shanghai Key Lab of Tuberculosis, Shanghai Pulmonary Hospital. All of them had no signs or symptoms of TB but tested positive by QFT-G (Qiagen, Germany). The LTBI group for diagnostic validation of the multiple-antigen combination set was recruited from individuals referred to the hospital suspecting but having no active TB clinical symptom and medical evaluation of LTBI based on a positive QFT-G. The active TB case was defined as a patient with all information regarding microbiological, pathological and radiological results and clinical response to anti-tuberculosis treatment according to WHO guidelines [21]. The negative control group included subjects with no history of TB and being negative by QFT-G. In addition, participants were excluded if: (i) they had HIV, hepatitis infections or autoimmune disorders and were taking any immunosuppressive medical treatment; (ii) blood samples had hemolytic reaction. More sample details regarding age, gender and sputum smear were listed in Additional file 1: Table S1.

### IFN-γ detection by QFT-G assay

The IFN-γ release assay was performed using the QFT-GIT assay kit as recommended by the manufacturer. Briefly, 1 ml of whole blood was collected from each subject by venipuncture directly into the three QFT blood collection tubes precoated with TB antigens (ESAT-6, CFP-10, and TB7.7), mitogen (positive control) or Nil (negative control), and incubated at 37 °C for 16 to 24 h. After centrifugation, the plasma samples can be stored for up to 28 days at 2 °C to 8 °C or, if harvested, below −20 °C for extended periods. The IFN-γ concentration was determined by a QFT Gold ELISA kit and the results were calculated using the manufacturer’s test software with the following criteria: the QFT results are interpreted as positive since the value of TB Antigen minus Nil [IU/mL] are more than 0.35 IU/mL when only nil & tb antigen tubes are used, or the value of TB Antigen minus Nil [IU/mL] are more than 0.35 IU/mL or Mitogen minus Nil [IU/mL] are more than 0.50 IU/mL when Nil, TB antigen and Mitogen tubes are used.

### Selection of GST-TBs

A total of 409 TB proteins, including 239 putative secreted proteins, 358 transmembrane proteins, 129 RD proteins and 91 latency-associated proteins, were predicted and screened from 3924 *M.*t*uberculosis* ORFs (open reading frames). The putative secreted and transmembrane proteins were predicted by bioinformatics tools, i.e. SignalP and TMHMM software, respectively. The RD proteins and the latency-associated proteins were chose from the relevant published data [16, 22, 23].

### Antibody assays using LTBI patient sera

IgG antibodies were detected by chemiluminescent ELISA as previously described [20]. Briefly, 100 μl pre-adsorbed sera (1:1000 dilution in PBST) was added to each well and incubated at 37 °C for 1 h. Each well was washed five times before 100 μl of 1:20,000 diluted HRP-conjugated anti-human IgG secondary antibody (Promega, USA) was added for incubation at 37 °C for a further 1 h. After washing five times, 100 μl of SuperSignal ELISA Femto Maximum Sensitivity Substrate solution (Pierce, USA) was then added. Between 1 and 5 min after adding the substrate, the bound antibodies were quantified by measuring the relative light units (RLUs) at 425 nm with a luminometer (SpectraMax M5, USA).

### Statistical analysis

Statistical analysis was done using SPSS Version 20 software (SPSS Inc., Chicago, IL, USA). Heat map was performed with R version 3.12 statistical software (The R Foundation for Statistical Computing, Vienna, Austria, available at http://www.r-project.org). Sensitivity and specificity were calculated according to the following formulas: sensitivity = number of true positives/(number of true positive + number of false negatives) and specificity = number of true negatives/(number of false positives + number of true negatives). A positive antibody test was defined as an RLU value greater than the cutoff value, i.e., the mean RLU value plus three SD from the negative healthy control serum.

## Results

### Screening of 409 GST-TB proteins with serum samples from LTBI individuals

**Fig. 1:**
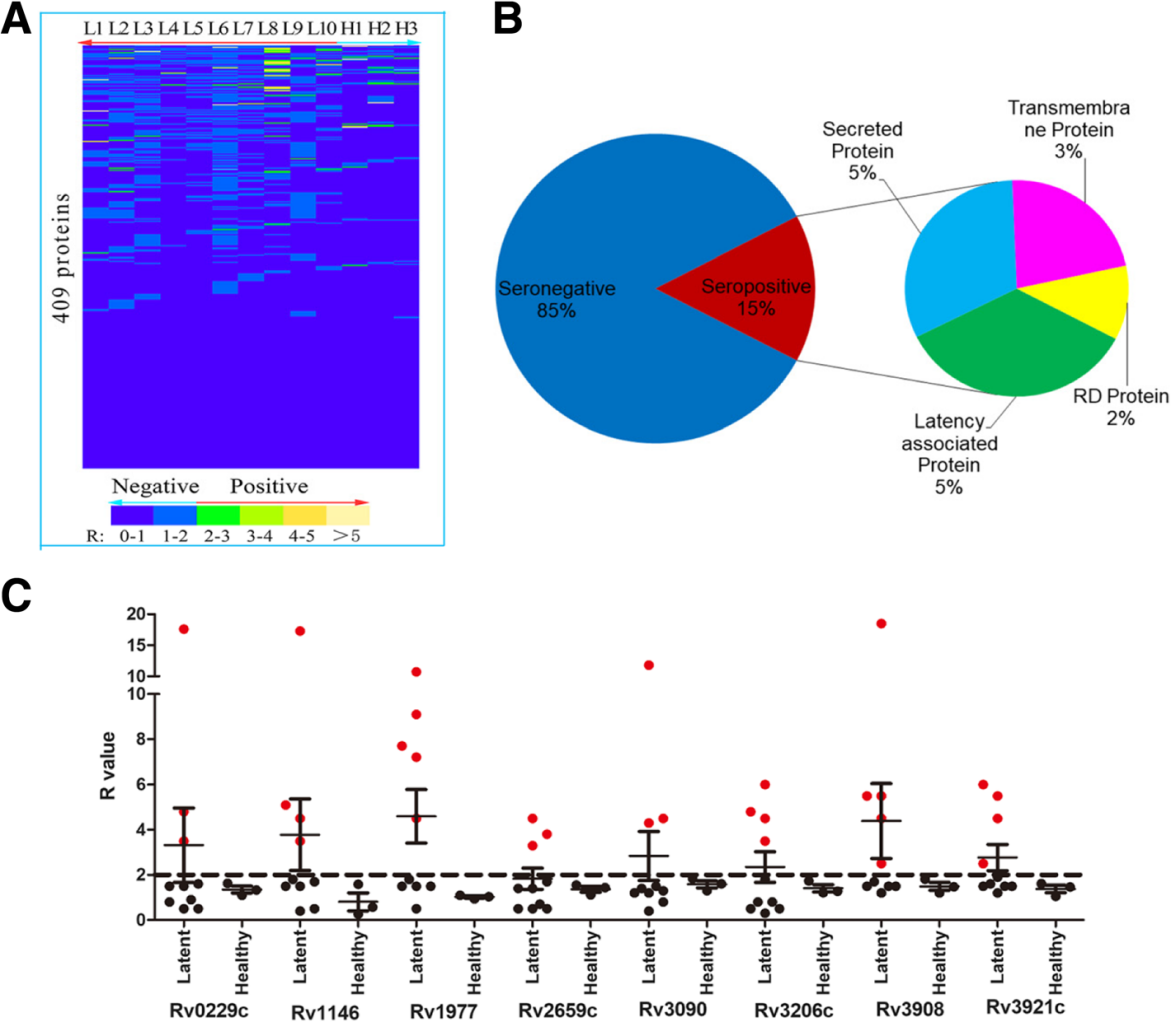
Screening of the GST-TBs with serum samples from latent tuberculosis infected (LTBI) individuals. **a** Serum from 10 LTBI individuals and three healthy controls was used for initial screening. Of 409 GST-TBs, 63 had seropositive reactions, defining the immuno-ORFeome of LTBI. Within the immuno-ORFeome, the rarely hit targets were predominantly latency-associated proteins and secreted proteins (**b**), while the frequently recognized antigens tended to be membrane-associated proteins (**c**). Five of eight highly-reactive proteins, Rv0229c, Rv1146, Rv3090, Rv3206c and Rv3921c, were membrane-associated proteins. The ratio of the RLUs was calculated using the formula: R = (RLUs of GST-TB − RLUs of PBS)/(RLUs of GST − RLUs of PBS). GST-TBs with *R* ≥ 2 were considered to indicate seropositive reactions. Red dots indicated seropositive reactions with R ≥ 2 and black dots indicated seronegative reactions with R< 2. The bars indicated the mean value of each individual antigen. RLUs = relative light units,RD = region of difference

For initial screening of the GST-TB fusion protein array (the “GST-TBs”), we used 10 LTBI individual serum samples, plus three healthy individual serum samples as negative controls. The ratio of the RLUs observed for a human serum sample for the GST-TBs (or the GST control) was calculated using the formula: R = (RLUs of GST-TB − RLUs of PBS)/(RLUs of GST − RLUs of PBS). GST-TBs with *R* ≥ 2 were considered to indicate seropositive reactions. Of 409 GST-TBs, 63 displayed seropositive reactions (Fig. 1a; Additional file 1: Table S2), defining the immuno-ORFeomeof LTBI. Figure 1b shows the relative proportion of each type of putative protein identified. Within the immuno-ORFeome, the rarely-recognized targets (i.e. those that were only hit occasionally) were predominantly latency-associated proteins and secreted proteins (34.9% (22/63) and 31.7% (20/63), respectively), while the frequently-recognized antigens tended to be membrane-associated proteins (62.5% (5/8)). In all, of the frequently-recognized antigens, eight, including two latency-associated proteins (Rv2659c and Rv3908), five membrane-associated proteins (Rv0229c, Rv1146, Rv3090, Rv3206c and Rv3921c) and one RD protein (Rv1977), reacted strongly with at least three LTBI samples and showed no cross-reactivity with serum samples from healthy controls (Fig. 1c). All eight were novel identified diagnostic antigens and were selected as candidate protein markers for further analysis. The eight proteins (without the GST-tag) were expressed in *Escherichia coli* (Additional file 2: Figure S1). It was noteworthy that, when compared with the immuno-ORFeome of active TB [20], 57.1% (36/63) antigens were LTBI-specific while 42.9% (27/63) cross-reacted with active TB (Additional file 1: Table S3, Additional file 2: Figure S2). These LTBI-specific antigens remained to be developed further.

### Serological assessment of eight candidate antigens

**Fig. 2:**
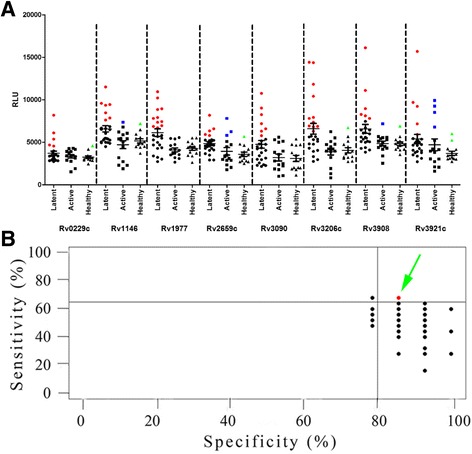
Assessment of eight candidate diagnostic antigens. **a** A total of 54 serum samples including 25 random latently TB-infected serum samples, 15 active infection samples and 14 healthy controls were used for detection. Red, blue and green dots represent serum samples in the LTBI, active TB and healthy control groups recognized by antigens, respectively. **b** The sensitivities and specificities of variable combinations of proteins (range 2–6 proteins) were calculated using the caret package in R statistical software. Each dot represents one combination (combinations with identical sensitivities and specificities are overlapping). Combinations with higher sensitivities and specificities (>0.7 and >0.8, respectively) to distinguish LTBI from healthy controls are marked with red dots. RLU = relative light units

A panel of 54 archived serum samples (25 latent infected samples, 15 active infected samples and 14 uninfected samples) was used to assess the potential of the eight candidate antigens as protein markers for the serodiagnosis of latent tuberculosis. As Fig. 2a and Additional file 1: Table S4 show, the specificity of all the recombinant proteins exceeded 80% (85.7%–100%), whereas their sensitivity ranged from 16.0% to 44.0%. However, two of the eight antigens, Rv2659c and Rv3921c, cross-reacted with active TB patient serum samples with sensitivities of 20.0% and 26.7%, respectively, indicating they could not be used to distinguish latent *M. tuberculosis* infection from active tuberculosis. The remaining six antigens, although highly specific, did not achieve a satisfactory serodiagnostic performance and were therefore assessed as part of multiple-antigen combinations.

A total of 57 different combinations, ranging from two individual antigens to six antigens, were prepared (Fig. 2b). Among these, the combination of four proteins – Rv1146, Rv1977, Rv3090 and Rv3206c – provided the best performance (68.0% sensitivity, 85.7% specificity) and was selected for further validation. Notably, all were novel antigens identified for LTBI serodiagnosis.

### Diagnostic validation of the multiple-antigen combination set

The sensitivity and specificity of the multiple-antigen combination was further analyzed for LTBI serodiagnosis using sera from 62 LTBI individuals and 32 healthy controls (Additional file 2: Figure S3; Table 1). Against these sera, all four antigens had a specificity of ≥93.8%, while the sensitivities of the four individual antigens varied from 32.3% to 43.6%. Of the infected serum samples, 66.1% (41/62) recognized at least one antigen; of these, only 7.3% (3/41) recognized all four antigens and 36.6% (15/41) reacted only with discrete antigens (two serum samples interacted with Rv1146 alone, six interacted with Rv1977, four with Rv3090, and three with Rv3206c). The combination of the four antigens increased the sensitivity significantly to 66.1%, with a specificity of 87.5% (Table 1).

**Table 1:** Sensitivities and specificities of four individual antigens and the multiple-antigen combination set with serum from LTBI individuals and healthy controls

Rv.	Latent TB (N = 62)			Sensitivity (%, 95% CI)	Healthy controls (*N* = 32)		Specificity (%, 95% CI)
	Specific Positive	Nonspecific Positive	Negative		Positive	Negative	
Rv1146	2	20	40	35.5 (24.0–48.7)	1	31	96.9 (82.0–99.8)
Rv1977	6	21	35	43.6 (31.2–56.7)	1	31	96.9 (82.0–99.8)
Rv3090	4	16	42	32.3 (21.3–45.5)	1	31	96.9 (82.0–99.8)
Rv3206c	3	19	40	35.5 (24.0–48.7)	2	30	93.8 (77.8–98.9)
Multiple	41		21	66.1 (52.9–77.4)	4	28	87.5 (70.1–95.1)

*Abbreviation: CI* confidence intervals. Specific Positive: serum samples which recognized only one of four antigens; Nonspecific Positive: serum samples which reacted with various antigens

## Discussion

Current strategies for LTBI diagnosis involve the traditional TST and the recent IGRAs. The TST consists of an intradermal injection of purified protein derivative (PPD) of tuberculin into the forearm. PPD is a crude and complex mixture of antigens, including those of *M. bovis*, *M. bovis* BCG and several species of nontuberculous mycobacteria (NTM), as well as *M. tuberculosis.* As a result, the specificity of the TST is limited due to cross-reactivity with previous BCG vaccination and NTM infection. In contrast, IGRAs measure T-cell release of IFN-γ in vitro after stimulation by specific *M. tuberculosis* antigens. The genes encoding these antigens, CFP-10, ESAT-6 and TB7.7, are all found in the RD of the *M. tuberculosis* genome, but not in *M. bovis*, *M. bovis* BCG or the majority of NTM, resulting in a higher specificity of the assay compared with the TST in populations with a high prevalence of BCG vaccination [24]. However, both tests have major disadvantages. The first is the poor positive predictive value for progression of LTBI to active disease. A systemic review concluded that there is little difference between TST and IGRA in this context [25], contributing to the WHO not endorsing the use of IGRAs in developing countries [4]. In addition, both of these immune-based tests perform with suboptimal sensitivity in individuals who are immunocompromised, and they are unable to discriminate active or past disease from latent infection. These factors underscore the need to identify more specific *M. tuberculosis* antigens and develop rapid, accurate, and cost-effective tests that can differentiate LTBI from patients with active tuberculosis and healthy individuals.

Granuloma, the hallmark of tuberculosis infection, is thought to limit bacterial growth via a variety of stress factors, including hypoxia, nutrient deprivation, low pH and NO, and drive *M. tuberculosis* into dormancy [15]. Several models mimicking dormancy revealed that during the latent stage, latency-associated proteins, including DosR “dormancy” regulons, transmembrane proteins and RD proteins are consistently upregulated for *M. tuberculosis* survival [14, 23, 26, 27]. Many latency-associated proteins have been confirmed to be antigens in TB immunity, but antigens for the serodiagnosis of latent *M. tuberculosis* are yet to be clearly identified [28]. Here, putative *M. tuberculosis* latency-associated proteins, transmembrane proteins, RD proteins and secreted proteins were explored for their serodiagnostic potential for LTBI.

While investigations of cellular immunity have focused on and been limited to a few typically immunodominant antigens, humoral immunity functions at the proteome scale. Recently, two proteome-wide approaches for screening of potential serodiagnostic antigens for active TB revealed that the subset of the proteome targeted by a human immune response was enriched for secreted and transmembrane proteins [29, 30], which was consistent with much of the earlier serological works [31, 32] and our previous study [20]. Some well-known immunodominant antigens*,* including ESAT-6, the 38 kDa antigen, MPT64 and HspX were identified. Kunnath-Velayudhan et al. further used macaque model and human serum samples to investigate proteome-scale antibody response during progression from latent Mycobacterium tuberculosis infection to active tuberculosis [33]. In this study, we aimed to identify novel diagnostic antigens using a recently developed high-throughput GST-fusion protein array technology. A total of 409 GST-TB fusion proteins were produced and interrogated with serum from LTBI individuals and healthy controls. The *M. tuberculosis* ORFeome was divided into three distinct sets based on the reactivity of sera: (i) the top eight frequently recognized antigens; (ii) additional frequently recognized antigens as well as proteins that were detected occasionally; and (iii) seronegative proteins. The first two sets of proteins (i + ii) define the immuno-ORFeome of latent *M. tuberculosis.* It is noteworthy that there was a significant increase (*P* = 0.03) in the proportion of latency-associated proteins among the latent TB immuno-ORFeome (34.9%) compared with that of active TB (19.8%) (Additional file 1: Table S5). This revealed that individuals with LTBI had a humoral response to the pathogen, thus it was reasonable to select LTBI serum samples to screen the GST-TB fusion protein library. On the other hand, we noticed that there were some LTBI-specific antigens when compared the immuno-ORFeome of LTBI with that of active TB, which deserve further to be investigated (Additional file 2: Figure S2). Of the eight highly reactive proteins, none were secreted proteins, two were latency-associated proteins, five membrane-associated proteins and one a RD protein. We hypothesize that, given the overall profile of the latency stage, secreted proteins were underrepresented because the number of metabolically active secreting bacilli was low and dormant mycobacteria did not secrete. However, transmembrane proteins, which may derive from live bacilli, dead bacilli or macrophage-secreted exosomes, could frequently interact with the immune system during latent infection. It was noteworthy that the drawbacks of this study still existed: 409 GST-TB fusion proteins were defined as *M. tuberculosis* ORFeome, which only constituted only a limited number (10%) of the total ORFs in *M. tuberculosis*. Furthermore, at the initial screening, only ten LTBI samples were used to interrogate the *M. tuberculosis* ORFeome, which may loss to identify some potential specific reactive antigens of LTBI. Although the majority of the frequently recognized targets would be successfully screened and some researchers successfully identify potential biomarkers for pulmonary tuberculosis at the first screening assay [30, 34], enough number of clinical samples interrogated would to the maximum extent guarantee to capture the reactive antigens [29, 35].

It is recognized that T-cell response to the typically immunodominant antigens, including ESAT-6 and CFP-10, cannot distinguish latent *M. tuberculosis* infection from active tuberculosis [36]. However, whether antibody response to any antigens could differentiate LTBI from active TB infection was less understood [37]. In this study, of the eight frequently recognized antigens, Rv2659c and Rv3921c cross-reacted with 20% and 26.7% of the active TB patient serum samples respectively (Fig. 2a), indicating they were incapable of distinguishing latent *M. tuberculosis* infection from active tuberculosis. The remaining six most reactive antigens could differentiate LTBI from active TB infection and healthy individuals. It was noteworthy that 3 to 6% of the healthy controls also reacted with the novel diagnostic antigens. The probable reasons for this false-positive reaction should also be explained. We took several steps to validate it: firstly, we examined whether other common human bacteria cross-reacted with *E. coli* to produce non-specific reactions, such as *Proteus bacillus vulgaris, Enterobacter aerogenes, Staphylococcus aureus and Staphylococcus albus* (data not shown). These bacterium supernatants were added into the serum samples to block nonspecific adsorption and compared the results. However, there was no significant difference between them. Moreover, in our previous study, we have found that the number and types of seropositive antigens vary from individual to individual, including healthy controls [38]. The person-to-person variations of antigen recognition may be linked to the genetic background of the people and thus have false-positive reactions with healthy controls. A multiple-antigen combination was required due to the unsatisfactory level of sensitivity of the individual antigens and the heterogeneity of the antibody response in LTBI individuals. The combination of four diagnostic proteins, Rv1146, Rv3090, Rv3206c and Rv1977, yielded a diagnostic sensitivity of 66.1% and specificity of 87.5%, which were significantly higher than those of the individual proteins and other combinations. Rv1146, Rv3090 and Rv3206c were membrane-associated proteins and Rv1977 was the RD protein; all of them were novel antigens. The multiple-antigen combination set provided a higher sensitivity and specificity and could be potentially developed as a novel serodiagnostic tool for LTBI.

## Conclusions

In this study, we used a recently developed high-throughput glutathione S-transferase (GST)-fusion technology to interrogate GST-TB fusion proteins using sera from LTBI and healthy individuals. Sixty-three reacted seropositive and six novel highly-reactive antigens were identified to have the potential to distinguish LTBI from active TB and healthy individuals. Furthermore, four diagnostic proteins were identified from them through analysis of various combinations and constructed as a multiple-antigen combination set for diagnosis of LTBI. A panel of 94 archived serum samples from LTBI and healthy individuals was used to validate the diagnostic performance of the multiple-antigen combination set that provided a higher sensitivity and specificity, which may be beneficial for tracking LTBI. The multiple-antigen combination set performed with great potential for the development of novel diagnostic tools for LTBI, which is important for the goal of global tuberculosis elimination.

## Additional files

Additional file 1: Table S1. Clinical characteristics of the study population; **Table S2**. The immunoproteome of latent tuberculosis; **Table S3**. The cross-reacted antigens between LTBI and active TB; **Table S4**. Proteins associated with latent tuberculosis; **Table S5**. Proportions of classes of protein in the immunoproteomes of latent and active TB. (DOC 126 kb)

Additional file 2: Figure S1. Purification of eight candidate proteins; **Figure S2**. Comparison of the immuno-ORFeome of LTBI with that of active TB; **Figure S3**. Diagnostic validation of the multiple-antigen combination set. (DOC 832 kb)

## Abbreviations

BCG: Bacilli Calmette-Guérin
GST: Glutathione S-transferase
IGRA: Interferon-γ release assays
LTBI: Latent tuberculosis infection
*M.* tuberculosis: Mycobacterium tuberculosis
NTM: Nontuberculous mycobacteria
ORF: Open reading frame
PPD: Purified protein derivative
RD: Region of difference
RLUs: Relative light units
TB: Tuberculosis
TST: Tuberculin skin test
