# Supplementary material for: ORFeome-based identification of biomarkers for serodiagnosis of Mycobacterium tuberculosis latent infection
Source: BMC Infect Dis. 2017 Dec 28;17:793. doi: 10.1186/s12879-017-2910-y (PMC5745629; doi:10.1186/s12879-017-2910-y)
Supplement: Supplementary file 1 — Clinical characteristics of the study population; Table S2. The immunoproteome of latent tuberculosis; Table S3. The cross-reacted antigens between LTBI and active TB; Table S4. Proteins associated with latent tuberculosis; Table S5. Proportions of classes of protein in the immunoproteomes of latent and active TB. (DOC 126 kb) [file 12879_2017_2910_MOESM1_ESM.doc]

**Table S1.** Clinical characteristics of the study population

| **Characteristic** | **Latent TB** | **Healthy control** | **Active TB** |
| --- | --- | --- | --- |
| **Round one** |  |  |  |
| Number | 10 | 3 | NA^#^ |
| Age, median (range) (yr) | 35.3 (25–47) | 39.3 (32–47) | NA |
| Male/female | 6/4 | 2/1 | NA |
| QFT-G assay+/− | 10/0 | 0/3 | NA |
| Sputum smear +/- | NA. | NA. | NA. |
| Period of enrollment (m, yr) | March, 2013 | | NA |
| **Round two** |  |  |  |
| Number | 25 | 14 | 15 |
| Age, median (range) (yr) | 43.9 (19–75) | 38.3 (24–72) | 42.7 (22–63) |
| Male/female | 16/9 | 9/5 | 10/5 |
| QFT-G assay+/− | 25/0 | 0/14 | 7/8 |
| Sputum smear +/- | NA. | NA. | 7/8 |
| Period of enrollment (m, yr) | August, 2013 – October, 2013 | | September, 2013 |
| **Round three** |  |  |  |
| Number | 62 | 32 | NA |
| Age, median (range) (yr) | 46.4 (19–-80) | 39.6 (9–74) | NA |
| Male/female | 34/28 | 19/13 | NA |
| QFT-G assay+/− | 62/0 | 0/32 | NA |
| Sputum smear +/- | NA. | NA. | NA. |
| Period of enrollment (m, yr) | January, 2014 – March, 2014 | | NA |

^#^Abbreviation: N.A., not applicable.

**Table S2.** The immunoproteome of latent tuberculosis

| Rv # | Gene | Annotation |
| --- | --- | --- |
| Rv0178 | - | Mce associated membrane protein |
| Rv0195 | - | two component transcriptional regulator |
| Rv0229c | - | hypothetical protein |
| Rv0288 | esxH | ESAT-6-like protein EsxH |
| Rv0309 | - | hypothetical protein |
| Rv0335c | PE6 | PE family protein PE6 |
| Rv0399c | lpqK | lipoprotein LpqK |
| Rv0432 | sodC | superoxide dismutase |
| Rv0436c | pssA | CDP-diacylglycerol--serine O-phosphatidyltransferase |
| Rv0455c | - | hypothetical protein |
| Rv0490 | senX3 | two component sensor histidine kinase SenX3 |
| Rv0569 | - | hypothetical protein |
| Rv0583c | lpqN | lipoprotein LpqN |
| Rv0640 | rplK | 50S ribosomal protein L11 |
| Rv0674 | - | hypothetical protein |
| Rv0677c | mmpS5 | membrane protein MmpS5 |
| Rv0700 | rpsJ | 30S ribosomal protein S10 |
| Rv0826 | - | hypothetical protein |
| Rv1100 | - | hypothetical protein |
| Rv1146 | - | transmembrane transport protein |
| Rv1152 | - | transcriptional regulator |
| Rv1284 | canA | beta-carbonic anhydrase |
| Rv1471 | trxB1 | thioredoxin |
| Rv1574 | - | phage protein |
| Rv1578c | - | phage protein |
| Rv1623c | cydA | cytochrome D ubiquinol oxidase subunit I CydA |
| Rv1793 | esxN | ESAT-6 like protein EsxN |
| Rv1805c | - | hypothetical protein |
| Rv1815 | - | hypothetical protein |
| Rv1881c | lppE | lipoprotein LppE |
| Rv1885c | lpqK | lipoprotein LpqK |
| Rv1906c | - | hypothetical protein |
| Rv1977 | - | hypothetical protein |
| Rv1987 | sodC | superoxide dismutase |
| Rv2012 |  | hypothetical protein |
| Rv2029c | pfkB | 6-phosphofructokinase PfkB |
| Rv2030c | - | hypothetical protein |
| Rv2107 | PE22 | PE family protein PE22 |
| Rv2194 | qcrC | ubiquinol-cytochrome C reductase cytochrome subunit C |
| Rv2659c | - | prophage integrase |
| Rv2660c | - | hypothetical protein |
| Rv2706c | - | hypothetical protein |
| Rv2875 | mpt70 | major secreted immunogenic protein Mpt70 |
| Rv3004 | cfp6 | low molecular weight protein antigen 6 |
| Rv3090 | - | hypothetical protein |
| Rv3130c-D1 | tgs1 | diacyglycerol O-acyltransferase |
| Rv3130c-D2 | tgs1 | diacyglycerol O-acyltransferase |
| Rv3134c | - | universal stress protein |
| Rv3206c | moeB1 | adenylyltransferase/sulfurtransferase |
| Rv3239c | - | transmembrane transport protein |
| Rv3267 | - | hypothetical protein |
| Rv3291c-D1 | lrpA | transcriptional regulator LrpA |
| Rv3291c –D2 | lrpA | transcriptional regulator LrpA |
| Rv3491 | - | hypothetical protein |
| Rv3627c | - | hypothetical protein |
| Rv3693 | - | membrane protein |
| Rv3737 | - | transmembrane protein |
| Rv3804c | fbpA | diacylglycerol acyltransferase/mycolyltransferase Ag85A |
| Rv3849 | espR | ESX-1 transcriptional regulator EspR |
| Rv3852 | hns | histone-like protein Hns |
| Rv3875 | esxA | ESAT-6 protein EsxA |
| Rv3908 | mutT4 | mutator protein MutT |
| Rv3921c | YidC | membrane protein insertase YidC |

**Table S3. The cross-reacted antigens between LTBI and active TB**

| Rv # | Gene | Annotation |
| --- | --- | --- |
| Rv0309 | - | hypothetical protein |
| Rv0432 | sodC | superoxide dismutase |
| Rv0436c | pssA | CDP-diacylglycerol--serine O-phosphatidyltransferase |
| Rv0490 | senX3 | two component sensor histidine kinase SenX3 |
| Rv0674 | - | hypothetical protein |
| Rv1146 | - | transmembrane transport protein |
| Rv1471 | trxB1 | thioredoxin |
| Rv1574 | - | phage protein |
| Rv1578c | - | phage protein |
| Rv1623c | cydA | cytochrome D ubiquinol oxidase subunit I CydA |
| Rv1815 | - | hypothetical protein |
| Rv1885c | lpqK | lipoprotein LpqK |
| Rv1906c | - | hypothetical protein |
| Rv1977 | - | hypothetical protein |
| Rv1987 | sodC | superoxide dismutase |
| Rv2029c | pfkB | 6-phosphofructokinase PfkB |
| Rv2194 | qcrC | ubiquinol-cytochrome C reductase cytochrome subunit C |
| Rv2659c | - | prophage integrase |
| Rv3130c-D2 | tgs1 | diacyglycerol O-acyltransferase |
| Rv3206c | moeB1 | adenylyltransferase/sulfurtransferase |
| Rv3291c | lrpA | transcriptional regulator LrpA |
| Rv3804c | fbpA | diacylglycerol acyltransferase/mycolyltransferase Ag85A |
| Rv3852 | hns | histone-like protein Hns |
| Rv3875 | esxA | ESAT-6 protein EsxA |
| Rv3908 | mutT4 | mutator protein MutT |
| Rv3921c | YidC | membrane protein insertase YidC |

**Table S4.** Proteins associated with latent tuberculosis

| Rv. | Gene | Sensitivity  (%, 95% CI) | Specificity  (%, 95% CI) | Annotation |
| --- | --- | --- | --- | --- |
| Rv0229c | - | 16.0 (5.3 - 36.9) | 92.9 (64.2 - 99.6) | hypothetical protein |
| Rv1146 | - | 28.0 (12.9 - 49.6) | 92.9 (64.2 - 99.6) | transmembrane transport protein |
| Rv1977 | - | 44.0 (25.0 - 64.7) | 100 (73.2 - 100) | hypothetical protein |
| Rv2659c | - | 20.0 (7.6 -41.3) | 92.9 (64.2 - 99.6) | prophage integrase |
| Rv3090 | - | 28.0 (12.9 - 49.6) | 100 (73.2, - 100) | hypothetical protein |
| Rv3206c | moeB1 | 40.0 (21.8 - 61.1) | 92.9 (64.2 - 99.6) | adenylyltransferase/sulfurtransferase |
| Rv3908 | mutT4 | 32.0 (15.7 - 53.6) | 92.9 (64.2 - 99.6) | mutator protein MutT |
| Rv3921c | - | 20.0 (7.6 -41.3) | 85.7 (56.2 - 97.5) | membrane protein insertase YidC |
| Multiple-antigen | - | 68.0 (46.5 - 84.3) | 85.7 (56.2 - 97.5) |  |

**Table S5. Proportions of classes of protein in the immunoproteomes of latent and active TB**

| **Seropositive proteins** | **Active TB** | **Latent TB** | ***P*-value** |
| --- | --- | --- | --- |
| Secreted proteins | 36 (39.1%) | 20 (31.7%) | 0.35 |
| Transmembrane proteins | 28 (30.4%) | 14 (22.2%) | 0.26 |
| Region of difference proteins | 10 (10.9%) | 7 (11.1%) | 0.96 |
| Latent-associated proteins | 18 (19.6%) | 22 (34.9%) | 0.03* |
| Total | 92 (100%) | 63 (100%) |  |

*P < 0.05 when compared by Pearson Chi-Square test.
