# Supplementary material for: ORFeome-based identification of biomarkers for serodiagnosis of Mycobacterium tuberculosis latent infection
Source: BMC Infect Dis. 2017 Dec 28;17:793. doi: 10.1186/s12879-017-2910-y (PMC5745629; doi:10.1186/s12879-017-2910-y)
Supplement: Supplementary file 2 — Purification of eight candidate proteins; Figure S2. Comparison of the immuno-ORFeome of LTBI with that of active TB; Figure S3. Diagnostic validation of the multiple-antigen combination set. (DOC 832 kb) [file 12879_2017_2910_MOESM2_ESM.doc]

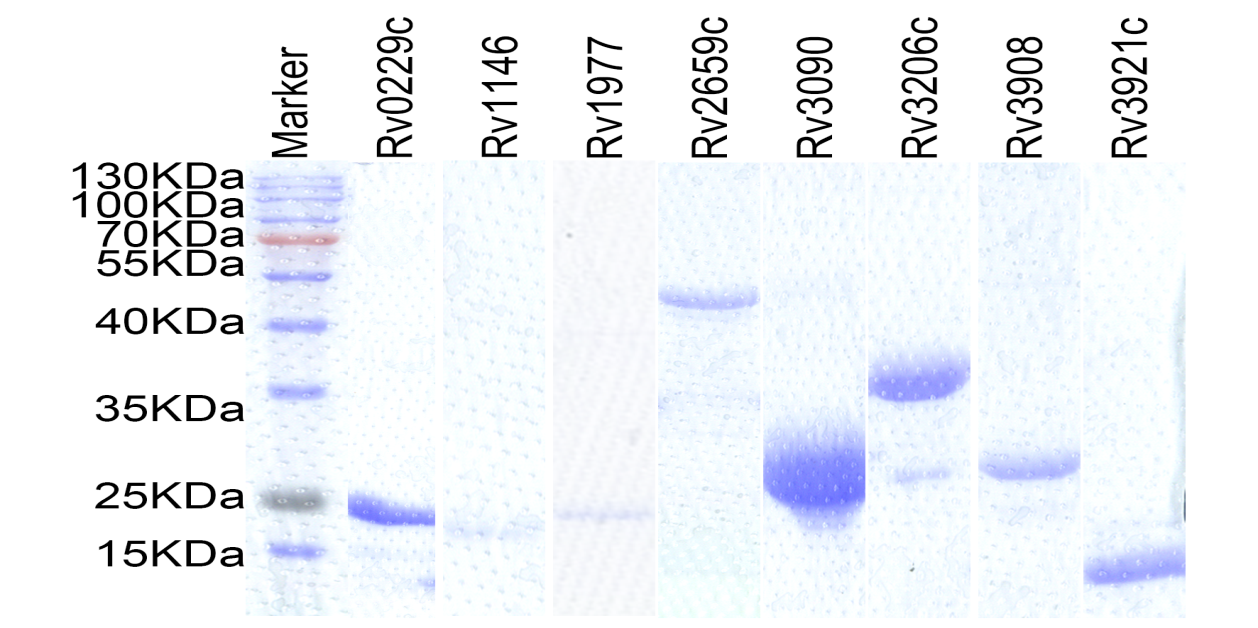


**Figure S1:** Purification of eight candidate proteins. The ORFs of the targeted proteins were amplified by PCR and cloned into the vector pET-28a. The proteins with the His-tag were re-produced in *E. coli*. Proteins expression in *E. coli Rosetta* (DE3; Novagen, Germany) was induced with 1 mM isopropyl-β-D-thiogalactoside (IPTG) and analyzed by sodium dodecyl sulfate polyacrylamide gel electrophoresis (SDS-PAGE) and Western blotting. When expressed in *E. coli*, some of those proteins were mainly expressed in the supernatant, while the others were predominately expressed in inclusion bodies. The inclusion bodies were denatured by the addition of 0.05 mM TCEP and 4.4% N-lauroylsarcosine and refolded in a universal refolding buffer C7 (1.0 mM TCEP, 250 mM NaCl, 12.5 mM β-cyclodextrin, 0.5 M L-arginine, 50 mM Tris-HCl pH 7.5) from the iFOLD Protein Refolding System 1 as described previously. Ni Sepharose 6 fast flow (GE Healthycare, USA) was used to purify the supernatant and refolded inclusion bodies of proteins. Purified recombinant proteins were then examined by SDS-PAGE and quantified through BCA protein quantitation kits. Quality One software was used to determine the purities of each protein. For the membrane proteins, as we chose only the largest extracellular domain for expression, the actual molecular weights of those proteins, including Rv0229c, Rv1146, Rv1977, Rv3090 and Rv3206c, were less than their theoretical values. The gels were cropped and run under the same experimental conditions.

**
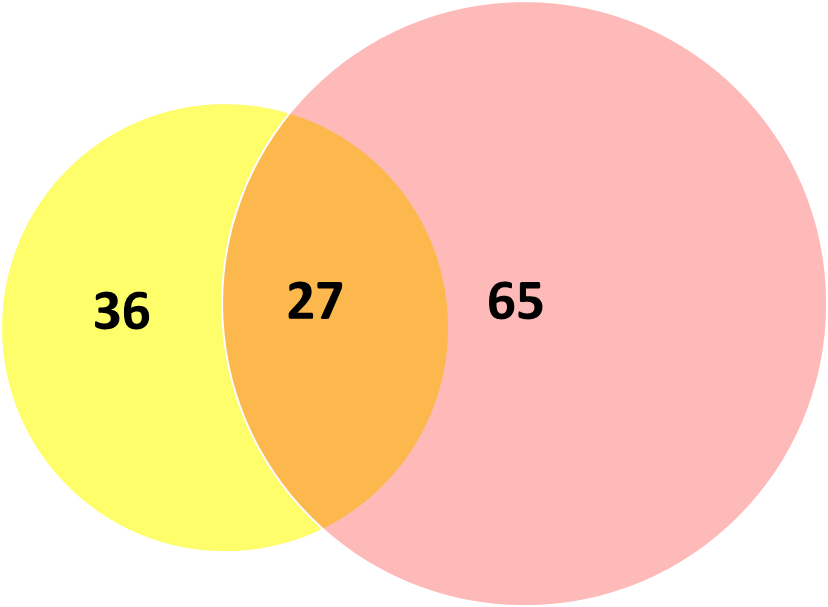
**

**Figure S2:** Comparison of the immuno-ORFeome of LTBI with that of active TB. Of seropositive reactions, 57.1% (36/63) antigens were LTBI-specific while 42.9% (27/63) cross-reacted with active TB. Of 92 seropositive reactions, 70.7% (65/92) antigens were active TB-specific while 29.3% (27/92) cross-reacted with LTBI. Yellow round indicated the immuno-ORFeome of LTBI, pink round indicated the immuno-ORFeome of active TB; and orange round indicated the cross-reacted antigens between them.


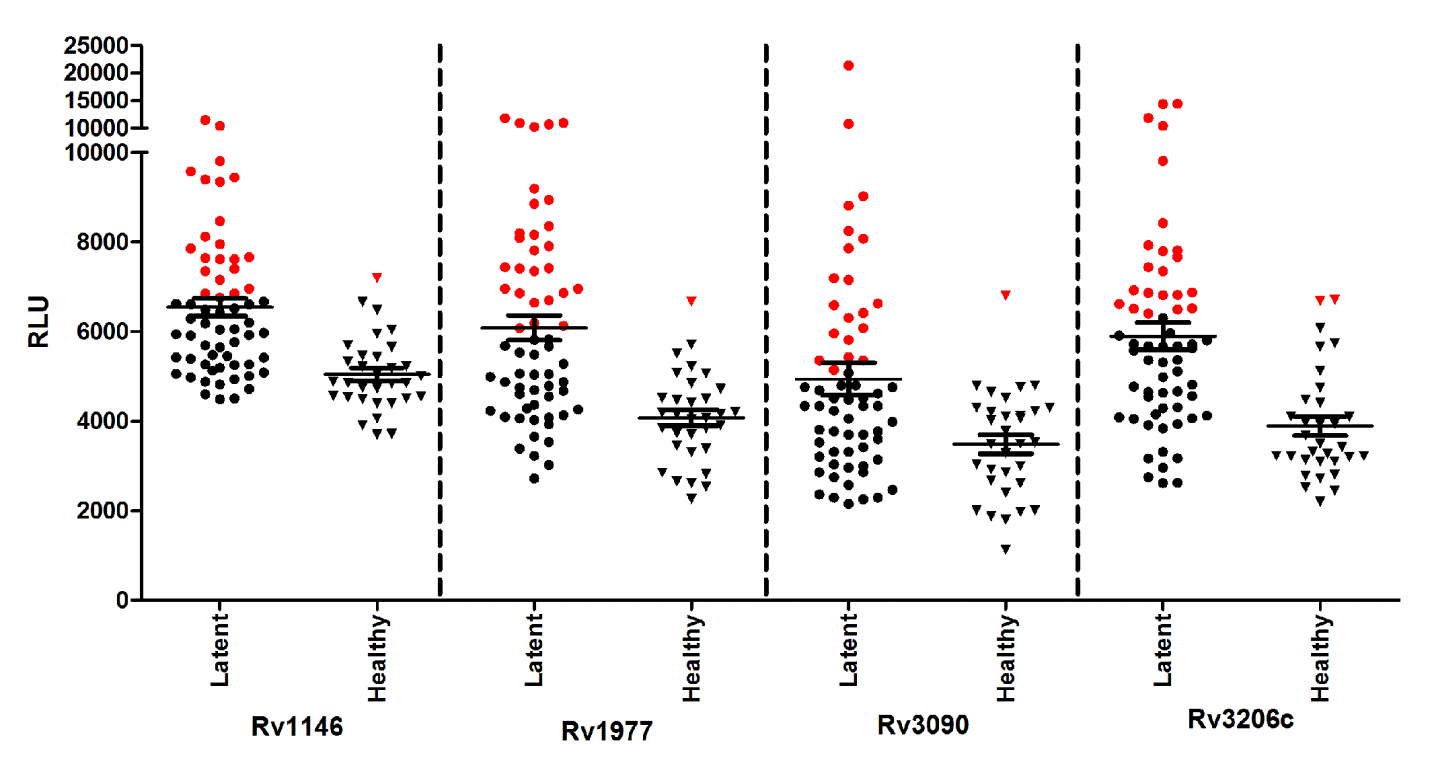


**Figure S3:** Diagnostic validation of the multiple-antigen combination set. Antibody responses to individual antigens of the combination set are displayed. Red dots represent antigens recognized by serum samples. RLU = relative light units.

**Reference**

1. Xu X, Zhang Y, Lin D, Zhang J, Xu J, Liu YM, Hu F, Qing X, Xia C, Pan W: **Serodiagnosis of Schistosoma japonicum infection: genome-wide identification of a protein marker, and assessment of its diagnostic validity in a field study in China**. *Lancet Infect Dis* 2014, **14**(6):489-497.

2. Zhou F, Xu X, Wu S, Cui X, Fan L, Pan W: **Protein array identification of protein markers for serodiagnosis of Mycobacterium tuberculosis infection**. *Sci Rep* 2015, **5**:15349.
